# Dr. Charles Lester Leonard

**Published:** 1913-11

**Authors:** 


The long list of martyrs to science is increased by the death of Dr. Charles Lester Leonard, a classmate of ours at the University of Pennsylvania in 1889, and one of the earliest Roentgenologists of Philadelphia. His death occurred at Atlantic City, September 22, from cancer, after a long period of suffering, with many operations undertaken in vain to remove the disease. He was 52 years old.

